# Three‐dimensional‐printed customized prosthesis for pubic defect: clinical outcomes in 5 cases at a mean follow‐up of 24 months

**DOI:** 10.1186/s12891-021-04294-6

**Published:** 2021-04-30

**Authors:** Yuqi Zhang, Li Min, Minxun Lu, Jie Wang, Yitian Wang, Yi Luo, Yong Zhou, Hong Duan, Chongqi Tu

**Affiliations:** 1 Department of Orthopedics, Orthopedic Research Institute, West China Hospital, Sichuan University, No. 37 Guoxuexiang, 610041 Chengdu, Sichuan People’s Republic of China; 2 Bone and Joint 3D-Printing & Biomechanical Laboratory, Department of Orthopedics, West China Hospital, Sichuan University, No. 37 Guoxuexiang, Sichuan 610041 Chengdu, People’s Republic of China

**Keywords:** 3D-printed, Prostheses, Type III hemipelvectomy, Short‐term outcomes

## Abstract

**Background:**

Pubic defects resulting from type III hemipelvectomy are commonly not reconstructed due to the need to preserve the weight-bearing axis. However, the opening of the anterior pelvic ring will inevitably lead to increased pelvic instability. To improve long-term pelvic stability, three-dimensional (3D)-printed customized prostheses were designed to reconstruct pubic defects. This study presents and evaluates the short-term clinical outcomes and complications from the use of this construct.

**Methods:**

Five patients who underwent type III hemipelvectomy and 3D-printed customized prosthesis reconstruction at our institution between 2017 and 2019 were retrospectively analysed. Operation time and blood loss during the operation were recorded. Local and functional recovery was assessed. Prosthetic position and osseointegration were evaluated. Oncology results and complications were recorded.

**Results:**

The prostheses consisted of three with stems and two without. The mean follow-up time was 23.6 months. At the last follow-up, all five patients were alive with no evidence of disease. No deep infections or local recurrence had occurred. The mean blood loss and mean intraoperative time were 1680 ml and 294 min, respectively. The mean functional MSTS score at the final follow-up was 29.8. Fretting wear around the prosthetic stem was found in 3 patients, while bone wear on the normal-side pubis was found in 2 patients. Osseointegration was observed in all patients.

**Conclusions:**

3D-printed customized prostheses for reconstructing pubic bone defects after type III hemipelvectomy showed acceptable early outcomes. The good outcomes were inseparable from the precision prosthesis design, strict surgical procedures, and sensible postoperative management.

## Introduction

Surgical reconstruction of pelvic bone tumour defects is a complicated procedure due to the complexity and irregularity of the pelvis. According to the Enneking and Dunham classification [1], type III hemipelvectomy involves resection of either part of or the entire pubis from the symphysis to the lateral margin of the obturator foramen. Nevertheless, this type of hemipelvectomy is uncommon, accounting for only approximately 11 % of procedures [2–4]. Most defects following type III hemipelvectomy are commonly not reconstructed because of the need to preserve the weight-bearing axis [4–6].

According to Tile [7], the anterior pelvic ring structure and the posterior ring structure account for 40 and 60 % of the stability of the entire pelvic ring, respectively, and the importance of the anterior pelvic ring should not be ignored. The biomechanical consequences of pubic symphysis resection include an increase in shear forces and vertical tension on the sacroiliac joint [8]. Loss of the pubis could lead to an increase in force, resulting in joint hypermobility and osteoarthritis [9]. In addition, hernia is frequently reported as a late complication in patients without reconstruction after type III hemipelvectomy [10, 11]. First, bony reconstruction provides an anchor for mesh and suture attachments, which adds to the integrity of the pelvic floor soft tissue reconstruction. Therefore, some surgeons who do not reconstruct bone defects still attempt pelvic floor repair [12]. Second, previous research has shown that patients develop stress fractures without reconstruction after pubis removal because the residual pelvis becomes unstable and distorted during walking and running [6]. Therefore, the integrity of the anterior pelvic ring should be valued while preserving the continuity of the weight-bearing axis.

Currently, some reconstruction methods have been reported after type III hemipelvectomy (Table 1). Mesh repair, artificial ligament repair, and allografting are the main reconstruction choices after type III hemipelvectomy [13, 14]. However, a large number of studies have revealed a variety of limitations with these reconstruction options. Although allografts provide good bone reconstruction, a high infection rate has been frequently reported in some studies [2]. In some studies, the allograft infection rate after type III hemipelvectomy reached as high as 20 % [14, 15]. Soft tissue reconstruction, such as mesh or artificial ligament repair, is easy and convenient, is commonly used to reconstruct bone defects and can effectively prevent incisional hernias [2, 12, 15]. However, pelvic mechanical stability is often ignored, which causes changes in pelvic structure and mechanics, resulting in complications such as acetabular shift and sacroiliitis. On the other hand, prostheses for pelvis reconstruction have good initial stability, early weight-bearing, and relatively rapid functional restoration but have not been applied in reconstruction after type III resection.

**Table 1:** Recent reconstruction ways after type III resection

First author	Year	Type of reconstruction	Number of cases	Follow-up time	MSTS
King LA [16]	1989	No reconstruction	12	0.75-15years	N/A^a^
Reddy SS [12]	2012	Marlex mesh	8	9.5years(average)	N/A
Sherman CE [4]	2011	No reconstruction	8	N/A	N/A
Arkoulis N [10]	2012	No reconstruction	1	N/A	N/A
Imanishi J [5]	2015	No reconstruction, fascia lata	2	N/A	100
Freitas RR [17]	2015	Fibular graft	2	N/A	N/A
		No reconstruction	3	N/A	N/A
Chao AH [2]	2015	Mesh, Soft tissue flap	14	N/A	N/A
Karim, S.M. [14]	2015	Allograft	5	0.58-6years	N/A
Zang J [15]	2018	LARS ligament	25	1.33-4years	88
Ene R [18]	2018	No reconstruction	1	N/A	N/A

^a^N/A means not available

Three-dimensional (3D)-printed customized prostheses with porous surfaces might be a reasonable solution for treating irregular bone defects, especially those of the pelvis. Currently, 3D-printed customized prostheses of the pelvis are mainly applied following hemipelvectomy, including type I and type II hemipelvectomy with or without partial pubis [19, 20]. However, no 3D-printed customized prostheses have yet been developed for bone reconstruction after pure type III hemipelvectomy.

We recently designed 3D-printed customized prostheses and applied them in the treatment of patients with malignancies involving region III, and a satisfactory outcome was observed. This study introduces our experience in using 3D-printed customized prostheses for reconstruction after type III hemipelvectomy and evaluates the clinical outcomes and associated complications with the use of 3D-printed customized prostheses for reconstruction.

## Materials and methods

### Patients

Five patients who received three-dimensional-printed customized prosthesis reconstruction after type III hemipelvectomy at our institution between June 2017 and February 2019 were retrospectively analysed in this study. The mean age of the patients was 36.6 years (range, 26–46 years) at the time of surgery. Three patients underwent superior pubic ramus resection, while two patients underwent total pubis resection.

**Fig. 1:**
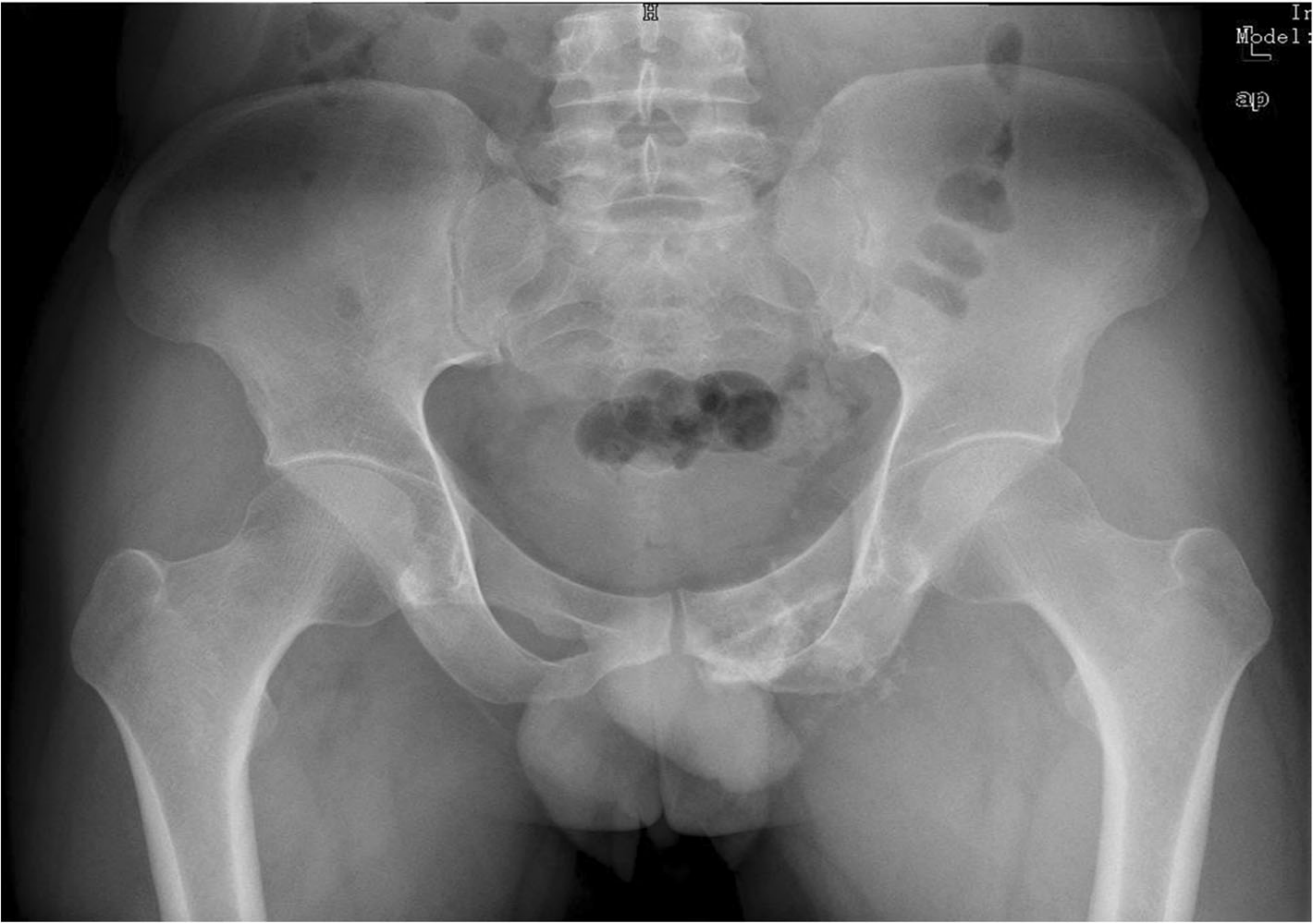
Pelvic X-ray in a patient with a chondrosarcoma (after biopsy) involving the superior and inferior pubis

To determine local disease and assess resectability, all patients underwent three-dimensional computerized tomography (3D-CT), magnetic resonance imaging (MRI), single-photon emission computed tomography (SPECT), or positron emission tomography/computerized tomography (PET/CT) and preoperative biopsy (Fig. 1). All patients were diagnosed with chondrosarcoma. Detailed patient characteristics are summarized in Table 2. Musculoskeletal Tumor Society (MSTS) scores were evaluated preoperatively. This rating scale is based on seven items, including pain, strength, range of motion, joint deformity, joint stability, emotional acceptance, and overall function. Each item is scored from 0 to 5 with a maximum possible score of 35.

**Table 2:** The demographics of the 5 patients treated with 3D-printed customized prosthesis

Case	Gender	Tumor location	Diagnosis	Follow-up (months)	IOT^a^ (min)	Blood Loss (ml)	MSTS		Oncologic outcome	Complications
							Pre.	Post.		
1	M	Entire pubis	Chondrosarcoma	16	290	1100	29	29	NED^b^	ED^c^
2	F	Entire pubis	Chondrosarcoma	18	180	300	31	29	NED	-
3	M	Pubic superioris	Chondrosarcoma	25	370	2500	31	31	NED	-
4	M	Pubic superioris	Chondrosarcoma	27	430	3700	33	31	NED	-
5	F	Pubic superioris	Chondrosarcoma	32	200	800	31	29	NED	-
mean	-	-	-	23.6	294	1680	31	29.8	-	-

^a^*IOT* Intraoperative time

^b^*NED* No evidence of disease

^c^*ED* Erectile dysfunction

 This study was approved by the Ethical Committee of our institution. Written informed consent to participate in this study was obtained from all the patients.

### Prosthesis design and fabrication

**Fig. 2:**
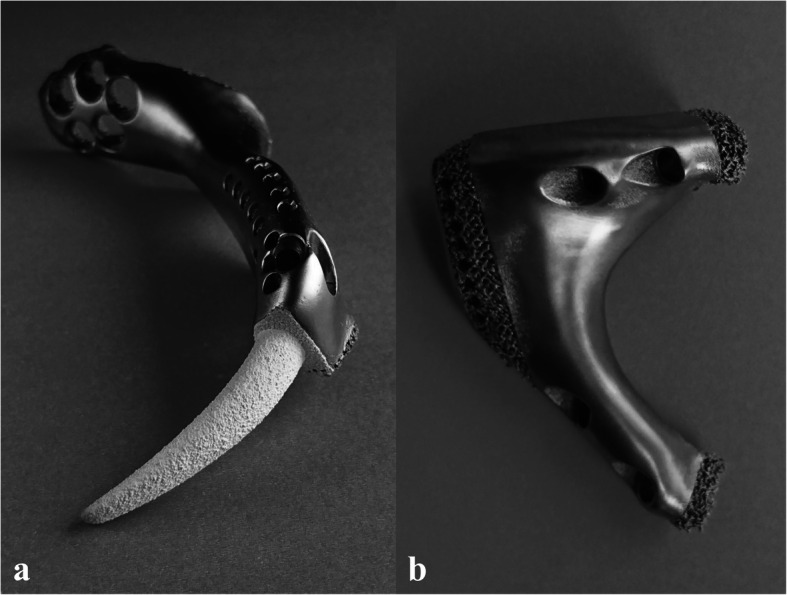
Prosthesis with stem (**a**) and prosthesis without stem (**b**)

All prostheses were designed by our clinical team and fabricated by Chunli Co., Ltd., Tongzhou, Beijing, China. Three-dimensional CT files were imported to Mimics V20.0 software (Materialise Corp., Leuven, Belgium) to build three-dimensional tumour and pelvis models. The tumour margin was determined by a combination of MRI, SPECT, and CT images based on a 3D model. Then, the osteotomy plane was obtained. According to the shape of the normal pelvis and the osteotomy plane, the preliminary shape of the prosthesis was designed by mirroring the corresponding normal part in Geomagic Studio software (Geomagic Inc., Morrisville, United States). After that, specific features, including suture holes and screw holes, were added to the prosthesis. Next, surface smoothing and unnecessary feature removal were performed. Osteotomy guides were designed at the same time. Finally, the porous structure was separated and generated in Magics V20 software (Materialise Corp., Leuven, Belgium). The porous structure included a 600 μm pore size and 70 % porosity (Fig. 2).

The prosthesis models were saved as stereolithography (STL) files and imported to Mimics to simulate implantation. Prostheses were fabricated by electron beam melting technology (ARCAM Q10plus, Mölndal, Sweden), and the osteotomy guides and the plastic trial model were fabricated with the stereolithography appearance technique (UnionTech Lite 450HD, Shanghai, China).

### Surgical techniques

All operations were performed by the same senior surgeon. The oblique supine lithotomy position and ilioinguinal incision were primarily used. Tumours were resected en bloc, and soft tissue was removed based on the results of the preoperative simulation. Osteotomies were performed with the help of osteotomy guides. Then, the plastic trial model the same size as the prosthesis was used first to confirm that the defect and the prosthesis matched perfectly. The next step was prosthesis implantation. Prosthesis fixation methods are different for different prostheses. For patients who underwent superior pubic ramus resection and reconstruction, the prosthesis stem was inserted into the normal pubis first. Then, the plate was fixed on the medial side of the pelvis with screws. For patients who underwent total pubis resection and reconstruction, the prosthesis was fixed first with screws and then tied to the contralateral pubis with a rivet string. Afterward, preserved muscles such as the musculus pectineus and musculus adductor longus were reconstructed before stitching. Intraoperative time and blood loss were recorded.

### Postoperative management

In our experience, although type III hemipelvectomy does not need arthroplasty, most adductor muscles and partial muscles around the obturator foramen were removed and reconstructed. To promote the recovery of soft tissue reconstruction and the integration of prosthesis and bone interface, bed rest for three-four weeks was undertaken for patients who received prosthesis with stem replacement. Furthermore, the hip joint’s proper postoperative movement could promote recovery and prevent thrombosis at the same time. Passive adduction and external rotation within 30° were allowed before week four and gradually changed to positive movement at week five. Meanwhile, hip joint movement with adductor relaxation and contraction was executed. Walking began at week five, and the weight-bearing of the affected limb increased gradually.

Besides, three-four days of bed rest was recommended for those patients who received prostheses without stem replacement. The hip joint and adductor relaxation movement and contraction were undergone with passive adduction and external rotation during the first week. Moreover, during the second week, walking with crutches with gradually increasing weight-bearing was undergone. The external rotation and positive abduction started in the third week. One month after surgery, patients could walk on flat ground without crutches.

All patients underwent a number of evaluations, including physical examination, pelvis X-ray, and tomosynthesis-shimadzu metal artefact reduction technology (T-SMART) of the pelvis regularly (monthly for the first three months and then trimonthly). Metastasis was evaluated by chest CT every six months. T-SMART was also used to evaluate osseointegration. The functional outcome was assessed by the Musculoskeletal Tumor Society (MSTS) score. Complications were recorded.

### Statistical analysis

Statistical analyses were performed using IBM SPSS Statistics software, version 22 (IBM SPSS, Armonk, NY). Descriptive statistics, including proportions and mean values, were calculated. The normality of continuous data was assessed with the one-sample Kolmogorov-Smirnov test. Preoperative and postoperative data were compared using the Wilcoxon signed-rank test. P < 0.05 was considered statistically significant.

## Results

Combined with postoperative histology results, the final pathology of all patients was chondrosarcoma. Two patients had grade II chondrosarcoma, and three patients had grade III chondrosarcoma. For tumour stage, according to the Enneking staging system, one patient had stage IIa disease, and four patients had stage IIb disease. All operations were R0 resections.

The mean blood loss was 1680 ml (range, 300 to 3700 ml), and the mean intraoperative time was 294 min (range, 180 to 430 min).

The mean follow-up period was 23.6 months (range, 16–32 months). At the time of the last follow-up, all five patients were alive with no evidence of disease (NED). No local recurrence was observed. The local surgical incision had healed well without infection or sinus tracts.

The mean functional MSTS score was 29.8 (range, 29–31), and the difference with the preoperative MSTS score was not significant. The VAS score improved from a median of 5 points (range 2 to 8) preoperatively to a median of 1 point (range 0 to 5, *p* = 0.001).

**Fig. 3:**
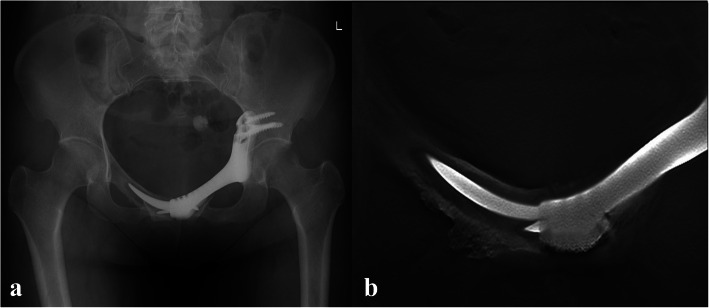
Six months after operation, X-ray (**a**) and T-SMART (**b**) showed fretting wear appeared around prosthetic stem

**Fig. 4:**
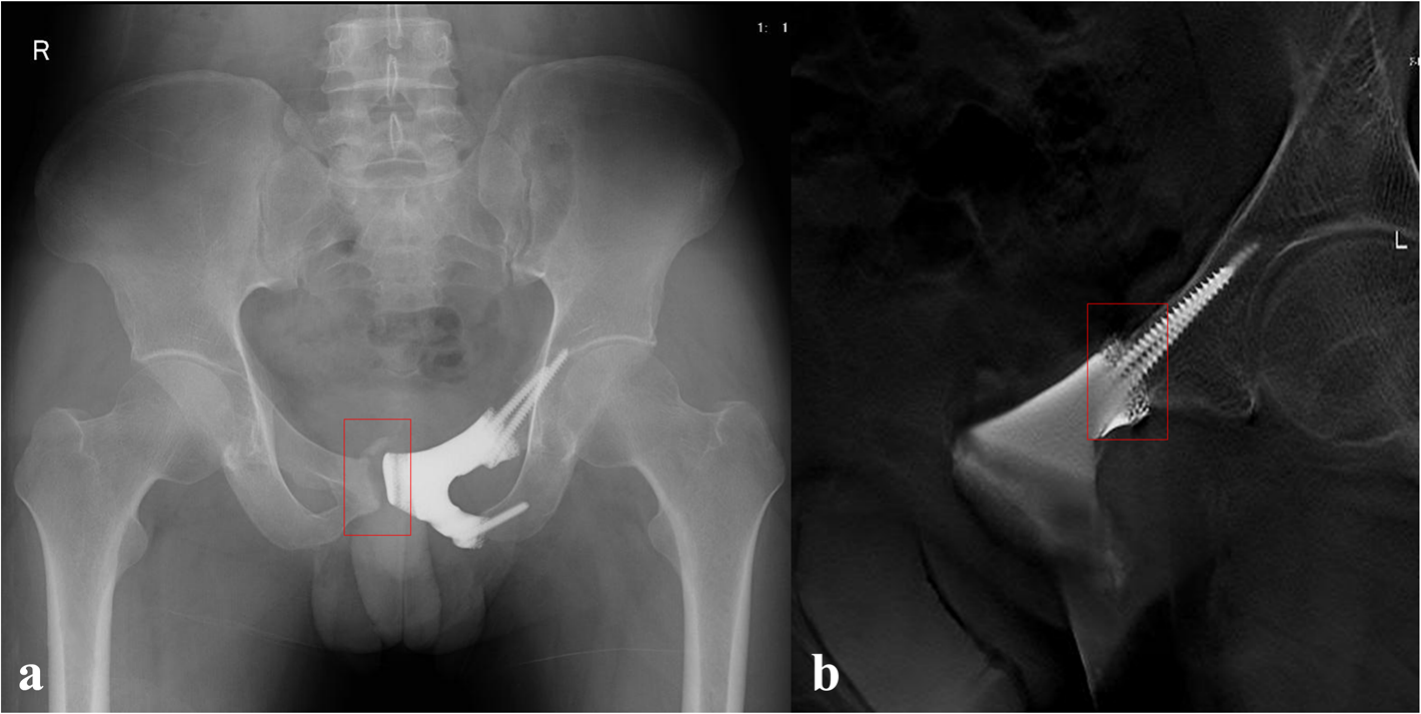
Four months after the operation, X-ray showed bone wear on the normal side pubis (**a**); Six months after operation, T-SMART showed preliminary osseointegration (**b**)

One male patient complained of erectile dysfunction after the operation, and functional recovery gradually occurred after five months. No dislocation or infection of the prosthesis was found through the last follow-up. Fretting wear around the prosthetic stem was found for the 3 patients who underwent superior pubic ramus resection and prosthesis with stem reconstruction six months postoperatively without any subjective discomfort (Fig. 3). Bone wear on the normal-side pubis was found for the 2 patients who underwent pubic ramus resection and prosthesis without stem reconstruction four months postoperatively without any subjective discomfort (Fig. 4a). T-SMART showed the absence of interfacial gaps between the prosthesis and bone six months postoperatively (Fig. 4b).

## Discussion

3D-printed customized prostheses are increasingly being used in pelvic defect reconstruction due to precise matching and have been reported to demonstrate good early results [21]. With its advantage of additive manufacturing, 3D-printed customized prostheses could add porous trabecular bone-like structures to promote bone ingrowth and bone-prosthesis interface integration, which play an essential role in bone defect reconstruction and repair. Previous studies showed that porous structures with a 300 to 800 μm pore size and 70 % porosity could enhance bone ingrowth [22–26]. Thus, porous structures with a 70 % porosity and a pore size of 600 μm were applied in this study, and good osseointegration was observed in the patients. Moreover, the precise forming technology of 3D printing allows 3D-printed prostheses to perfectly match the defect shape, which can reduce the difficulty of prosthesis implantation. However, conversely, it can increase the difficulty in intraoperative osteotomy, which means that osteotomy guide assistance and preoperative simulation are necessary.

In our series, acceptable functional results with an average MSTS score of 29.8 and an obvious VAS decrease were achieved, comparable with those of a previous study [15]. Favourable function depends on a good reconstruction, including bony reconstruction and soft tissue reconstruction. The intact pelvic ring was reconstructed to distribute stress evenly and improve the stability of the pelvis. In addition, the attachments of muscles were carefully reconstructed with sutures, providing durability for further rehabilitation. Sensible postoperative rehabilitation was another critical factor influencing functional rehabilitation after the operation.

Complications related to prostheses were observed in those patients who received superior pubic ramus resection and reconstruction. We believe this resulted from the mechanics of the pelvic ring and the prosthesis design. For these three patients, we designed a prosthesis with an arcuated stem fitting the normal-side pubic medullary cavity and fixed the prosthesis with plates and screws and by inserting the stem into the normal-side pubic medullary cavity. During the follow-up, we found fretting wear around the prosthetic stem, indicating that the prosthesis was not as stable as we had thought. We inferred that the pubic symphysis and the low-mobility sacroiliac joint resulted in the fretting wear. Because the pelvis is a closed ring and the low-mobility sacroiliac joint can affect the pubic symphysis, as long as the sacroiliac joint frets, so too does the pubic symphysis. Consequently, we designed a prosthesis without a stem to attempt to reconstruct the pubic symphysis. This procedure produced bone wear on the normal-side pubis instead of fretting wear because the elastic modulus was ignored. Direct contact between the alloy of the prosthesis and the bone can lead to bone wear. In our observation, however, this wear gradually stabilizes.

Compared with allograft reconstruction, 3D-printed customized prostheses significantly reduce infection rates. S Mohammed Karim et al. [14] reported allograft reconstruction in 14 patients after type III pelvectomy with an average follow-up of 35 months. At the last follow-up, only five patients were able to ambulate without an assistive device. Six patients had major complications, including hip instability in one, symptomatic hernia in one, dislocated total hip arthroplasty in one, infection in two, and graft failure in one. Mankin, H.J.’s research [27] showed that 2 of the 14 patients who underwent allograft reconstruction after partial pubic hemipelvectomy had infection, and 1 had hardware failure with nonunion. In our study, no dislocation or infection of the prosthesis was found through the last follow-up. Preoperative high-temperature sterilization of the prosthesis, intraoperative repeated pulse flushing, and iodophor immersion could be important for preventing infection. Multilayer stitching and postoperative wound care may be helpful for avoiding incision infection. Porous structures enhance bone ingrowth and increase the stability of the prosthesis.

Additionally, precise intraoperative osteotomy and proper prosthesis implantation are critical in shortening the surgery time and reducing bleeding. To improve the accuracy of the osteotomy and reduce exposure, osteotomy guides were applied during the operation. In addition to the osteotomy guides, a plastic trial model, slightly smaller than the prosthesis, was also used to ensure the matching of the prosthesis and the defect. Moreover, the order of prosthesis fixation is important for implantation. For prostheses with stems, the prosthesis stem must first be inserted into the medullary cavity, and then the prosthesis can be fixed to the acetabular side. For prostheses without stems, prosthesis fixation to the acetabular side must be performed first. Then, the pubis side can be reconstructed. Furthermore, suture holes were designed on the surface of the prosthesis to reconstruct related muscle attachments, which decreased the difficulty of soft tissue reconstruction and improved the efficiency of the procedure.

Erectile dysfunction (ED) was first reported in a type III hemipelvectomy. It has been reported that 3 % of ED cases may result from pelvic fractures or perineal blunt trauma [28]. It is assumed that such ED cases are due to lesions of the cavernous nerves or branches of the internal pudendal arteries, which pass in close proximity to the pelvic bones and posterior urethra [29]. Combined with the findings of this study, tumour resection involving the inferior pubic ramus could probably affect the branches of the internal pudendal arteries. To prevent ED after type III hemipelvectomy, resection of the inferior ramus of pubis should be performed carefully, especially when dealing with the medial side. The blood vessels and nerves in this area should be carefully protected and not stretched excessively. Although the symptoms were relieved after five months, surgeon should still take care to avoid this complication. Because of the small number of samples, it is unknown whether the nerve or artery was damaged and whether the normal-side nerve compensates or the damaged area gradually recovers after nerve traction injuries.

This study has some limitations. First, the oncologic outcome was not evaluated because of the small number of patients. Second, this study is a retrospective case series with a short-term follow-up; it is possible that more complications might arise over long-term follow-up. Moreover, due to the small sample size and the different extents of resection and disease processes, it is difficult to make a control group for comparison. Therefore, further study involving a multi-institutional sample is needed. In addition, we will continue to follow-up these patients.

## Conclusions

3D-printed customized prostheses for reconstructing pubic bone defects after type III hemipelvectomy showed acceptable early outcomes. The good outcomes were inseparable from the precision prosthesis design and strict surgical procedures. Despite the favourable outcomes, we found that 3D-printed customized prostheses without stems were more biomechanical and performed better than those with stems. The design of the prosthesis should be optimized, and long-term follow-up is required in future studies.

## Data Availability

The data and materials are available from the medical records department of the West China Hospital. The datasets used and analyzed during the current study are available from the corresponding author on reasonable request.

## Abbreviations

3D: Three dimensional
CT: Computerized tomography
MRI: Magnetic resonance imaging
SPECT: Single-photon emission computed tomography
PET/CT: Positron emission tomography/ computerized tomography
T-SMART: Tomosynthesis-shimadzu metal artefact reduction technology
MSTS: Musculoskeletal Tumor Society
NED: No evidence of disease
ED: Erectile dysfunction
